# Pulmonary Function in Adults With Type 2 Diabetes With and Without Obesity

**DOI:** 10.1016/j.chpulm.2023.100014

**Published:** 2023-09-03

**Authors:** Charles F. Hayfron-Benjamin, Ruth Korkor Tei, Josephine Korang Osei-Tutu, Tracy Amo-Nyarko, Patience Vormatu, Joana N. Ackam, Gloria Odom Asante, Latif Musah, Anastasia N.K. Bruce, Kwaku Amponsah Obeng

**Affiliations:** a Departments of Respiratory and Vascular Medicine, Amsterdam UMC, University of Amsterdam, Amsterdam, The Netherlands; b Department of Physiology, University of Ghana Medical School, Accra, Ghana; c Department of Anaesthesia, University of Ghana Medical School/Korle Bu Teaching Hospital, Accra, Ghana; d Department of Medicine and Therapeutics, Korle Bu Teaching Hospital, Accra, Ghana; e Department of Respiratory Therapy, University of Ghana Medical Centre, Accra, Ghana; f Department of Biomedical Sciences, University of Cape Coast, Accra, Ghana; g Family Health Medical School, Accra, Ghana; h School of Nursing, University of Ghana, Accra, Ghana

**Keywords:** obesity, obstructive airway disease, pulmonary dysfunction, restrictive lung disease, type 2 diabetes

## Abstract

**Background:**

Existing reports show a bidirectional association between type 2 diabetes (T2D) and pulmonary dysfunction. Obesity, which is causally related to both T2D and pulmonary dysfunction, could play an important role in this association. However, this has not been reported.

**Research Question:**

What are the associations of measures of obesity with pulmonary function in T2D?

**Study Design and Methods:**

This was a cross-sectional study among 464 adults with T2D. Spirometry was performed according to the American Thoracic Society/European Respiratory Society guidelines. The predicted values of the spirometric indices were determined using the Global Lung Function Initiative 2012 equations. The values of FEV_1_/FVC and FVC were used to categorize pulmonary function patterns as normal, obstructive, restrictive, or mixed. Waist circumference (WC) was measured at the midpoint between the lower margin of the lowest palpable rib and the top of the iliac crest.

**Results:**

The mean age, diabetes duration, and female/male ratio of the participants were 55.09 ± 10.45 years, 10.00 ± 7.36 years, and 2:1, respectively. In a multiple linear regression model, WC was a significant predictor of FVC (*P* = .018) and FEV_1_/FVC ratio (*P* = .005), but not FEV_1_ (*P* = .472). BMI was a significant predictor of FEV_1_/FVC ratio (*P* = .031), but not FEV_1_ (*P* = .802) or FVC (*P* = .129). In a multivariable logistic regression model adjusted for age, sex, socioeconomic status, diabetes duration, glycated hemoglobin, statin use, and smoking pack-years, increasing *z* score WC was associated with higher odds of restrictive spirometry (OR, 1.32; 95% CI, 1.05-1.66; *P* = .019) but not airway obstruction (OR, 0.65; 95% CI, 0.42-1.03; *P* = .067). There were no significant associations of increasing *z* score BMI with restrictive spirometry (OR, 1.24; 95% CI, 0.98-1.58; *P* = .075) or airway obstruction (OR, 0.79; 95% CI, 0.51-1.24; *P* = .305).

**Interpretation:**

Increasing WC is associated with restrictive spirometry, independent of conventional diabetes and pulmonary risk factors. Future research could explore the role of the reversal of central obesity on pulmonary function in T2D.

**Take-home Points**

**Study Question:** Is the relationship between type 2 diabetes and spirometric impairment explained by central obesity?

**Results:** After adjustment for conventional diabetes and pulmonary risk factors, increasing waist circumference was associated with higher odds of restrictive spirometry but not airway obstruction.

**Interpretation:** Increasing waist circumference is independently associated with restrictive spirometry but not airway obstruction.

There is a growing body of evidence linking type 2 diabetes (T2D) to pulmonary impairment, both epidemiologically and mechanistically.^1^^,^^2^ For example in the Fremantle diabetes study, individuals with T2D were observed to have lower lung volume and airflow limitation; airflow limitation was a predictor of death after adjusting for other recognized risk factors.^3^ The biological basis of pulmonary dysfunction in T2D remains uncertain. Metabolic processes in T2D include endothelial cell dysfunction from increased oxidative stress that results in the overexpression of vasoconstrictor mediators^4^ and elevated levels of advanced glycation end products from persistent hyperglycemia.^5^^,^^6^ The pulmonary vasculature and extracellular matrix cells express glucose transporter 1, enabling an unlimited glucose influx from the blood to the interstitium.^7^^,^^8^ It has therefore been postulated that these systemic vascular pathologic processes may affect the pulmonary systems. Chronic hyperglycemia may also result in abnormal regulation of inflammatory pathways, including exaggerated pulmonary inflammatory responses to airborne particles and tobacco smoking.^9^ However, these mechanisms do not sufficiently explain pulmonary dysfunction in T2D.^2^^,^^9^^,^^10^ Compounding the relationship, there is evidence supporting the view of reverse causation (ie, reduced pulmonary function results in T2D).^11^

One potential factor that may explain disparities in the existing reports linking pulmonary dysfunction and diabetes is obesity. In all ethnic groups, T2D is strongly associated with obesity.^12^ In the general population, WC is known to correlate inversely with measures of pulmonary mechanics including FEV_1_ and FVC, regardless of BMI; WC may negatively impact pulmonary mechanical function by limiting diaphragm expansion.^13^ Although both BMI and WC are usual measures of overweight/obesity, WC correlates better to intraabdominal and subcutaneous adipose tissue than BMI.^13^ Given these, the relationship between T2D and spirometric impairment may be explained by central obesity. We tested the hypothesis that in T2D, increasing WC is associated with reduced FEV_1_, FVC, and FEV_1_/FVC ratio.

## Study Design and Methods

### Study Design

Data from the study have been previously published.^14^^,^^15^ The study was a cross-sectional study among adult native Ghanaians with T2D managed at a National Diabetes Management and Research Center in Accra, Ghana. From 2019 to 2022, 500 eligible participants were recruited for pulmonary, cardiac, and vascular functional assessment. The participants were recruited via systematic sampling from patients who reported clinic visits during the study period. Eligible participants were patients with an established diagnosis of T2D without primary heart disease (primary myocardial disease) and/or previous or current heart failure. T2D diagnosis was based on fasting plasma glucose level ≥ 7.0 mmol/L and/or 2-h plasma glucose level ≥ 11.1 mmol/L and/or patients who were on hypoglycemic agents who reported the start of their diabetes at age > 30 years and whose diabetes initially did not require insulin for management. The current analyses included 464 participants aged ≥ 35 years with no primary lung disease, and who performed technically acceptable spirometry. Before the start of data collection, ethical approval was obtained from the ethics committees of our institutions (Nos. CHS-Et/M6-P2.14/2017-2018 and KBTH-IRB/000124/2019). All participants provided written informed consent before enrollment in the study.

### Measurements

Assessment of baseline characteristics has been previously described.^16^ Weight was measured in light clothing and without shoes with a Seca 877 scale (Seca). Height was measured without shoes with a Seca 217 stadiometer (Seca). BMI was calculated as weight divided by the square of height. All the anthropometric measurements were measured twice by the same assessor, and the average of the two measurements was used for analyses. Based on BMI, obesity was defined as BMI ≥ 30 kg/m^2^.^17^ Waist circumference (WC) was measured at the midpoint between the lower margin of the lowest palpable rib and the top of the iliac crest. Hip circumference was measured around the widest portion of the buttocks, at the level of the greater trochanters, with the tape parallel to the floor. Central obesity was defined as a WC > 102 cm in male participants and > 88 cm in female participants.^17^ The percentage of body fat and visceral body fat level was measured using the arm-leg bioimpedance technique using the Omron Body Composition BF-506 Monitor (Omron Healthcare, Inc).

### Spirometry Testing

Prebronchodilator spirometry was conducted by trained physicians/technicians using the Vitalograph Pneumotrac Portable Screening Pneumotachograph (Vitalograph Ltd) according to the American Thoracic Society/European Respiratory Society guidelines.^18^ The spirometer was calibrated daily. The spirometry procedure was first demonstrated to each participant, after which the participant performed spirometry. After the study participant assumed the correct posture and attachment of the nose clip, the participant inhaled completely and rapidly after which the mouthpiece was inserted into the participant’s mouth, with the lips sealed around the mouthpiece and the tongue not occluding the mouthpiece. The FVC maneuver was then started with minimal hesitation (within 1-2 s after inspiring to total lung capacity). The participant was prompted to blast the air from their lungs and was then encouraged to fully exhale until no more air could be expelled while maintaining an upright posture. Throughout the FVC maneuver, enthusiastic coaching of each participant was carried out using appropriate body language and phrases (eg, keep blowing, keep going). This maneuver was repeated until a minimum of three maneuvers (and no more than eight) and acceptable and repeatable results were obtained.

Measured and calculated spirometric indices included FEV_1_, FVC, and FEV_1_/FVC ratio. The predicted values of FEV_1_, FVC, and FEV_1_/FVC were determined for each participant using the Global Lung Function Initiative 2012 equations.^19^ Based on the American Thoracic Society recommendation, race-neutral average reference equations were used.^20^ Abnormal results for spirometric indices were determined by comparison to their lower limits of normal.^19^ The values of FEV_1_/FVC and FVC were used to categorize pulmonary function patterns as normal, obstructive, restrictive, or mixed obstructive and restrictive based on American Thoracic Society/European Respiratory Society guidelines.^21^ Because of heterogeneity in individuals with impaired spirometry, we analyzed FEV_1_, FVC, and FEV_1_/FVC as a continuous variable via *z* score. Reductions in FEV_1_, FVC, and FEV_1_/FVC are known to predict mortality and/or adverse cardiovascular events.^22^, ^23^, ^24^, ^25^, ^26^

### Statistical Analysis

Differences in clinical characteristics between individuals with and without obesity or central obesity were assessed by χ^2^ test or Fisher exact test for categorical variables, *t* test for continuous variables, or Mann-Whitney U test for variables not normally distributed. Visual displays of the relationships between measures of adiposity and spirometry were presented, after which Pearson correlation analyses were used to examine the linear associations of WC and BMI with FEV_1_, FVC, and FEV_1_/FVC ratio. After this, multiple linear regression models were constructed to evaluate associations of WC and BMI with FEV_1_, FVC, and FEV_1_/FVC ratio, with adjustment for potential covariates. Results were presented as beta value (95% CI). Multicollinearity was checked using correlation coefficients and variance inflation factor values. Cutoff values considered as significant multicollinearity were ≥ 0.8 for the correlation coefficient and/or ≥ 5 for variance inflation factor. A high correlation coefficient (0.8) was observed for BMI and WC. Therefore, separate models were run for them. Multivariate logistic regression analyses were used to examine the associations of increasing *z* score WC or BMI (independent variable) with restrictive spirometry or airway obstruction (dependent variable), with adjustment for potential covariates. Three models were used in the analyses. Model 1 was unadjusted. Model 2 was adjusted for age and sex. Model 3 was adjusted for age, sex, socioeconomic status, diabetes duration, glycated hemoglobin (HbA1c) concentration, statin use, and smoking pack-years. Statin use was adjusted in the fully adjusted model because previous studies in individuals with COPD have shown that long-term statin use reduces inflammatory factors including C-reactive protein and IL-6 and improves lung function indices including FEV_1_ and FEV_1_/FVC.^27^ ORs and their corresponding 95% CIs were estimated. A statistical test of significance was set at *P* < .05. Data were analyzed using IBM SPSS version 25 for Windows (SPSS Inc).

## Results

### General Characteristics

Table 1 describes the baseline characteristics of the study population. Although 54.1% of the study participants had central obesity based on elevated WC, 36.4% were obese based on elevated BMI. The proportion of individuals with obesity class 3 (BMI ≥ 40 kg/m^2^) was 5.6%. Compared with individuals without central obesity, individuals with central obesity were more frequently female, much older, and less educated, but less frequently tobacco users. Additionally, a higher proportion of individuals with central obesity used insulin and statins compared with those without central obesity. Mean age, diabetes duration, systolic BP, and pulse rate were higher in individuals with central obesity than those without central obesity. Mean HbA1c concentration and cholesterol concentration were similar between the two groups. Similar associations were observed when obesity was based on BMI > 30 kg/m^2^, except for the highest education level attained. WC was higher in the high BMI group than in the high WC group. Similarly, visceral fat was higher in the high BMI group than in the high WC group.

**Table 1:** General Characteristics of Study Participants

Variable	All Participants (N = 464)	Central Obesity (Based on WC)			Obesity (Based on BMI)		
		No (n = 213)	Yes (n = 251)	*P* Value	No (n = 295)	Yes (n = 169)	*P* Value
Characteristic							
Sex	. . .	. . .	. . .	< .001	. . .	. . .	< .001
Female	314 (67.7)	79 (37.1)	235 (93.6)	. . .	165 (55.9)	149 (88.2)	. . .
Male	150 (32.3)	134 (62.9)	16 (6.4)	. . .	130 (44.1)	20 (11.8)	. . .
Age, y	55.09 ± 10.45	52.95 ± 11.33	56.90 ± 9.28	< .001	54.24 ± 10.89	56.58 ± 9.49	.016
Lower education	196 (56.6)	66 (47.8)	130 (62.5)	.007	111 (54.4)	85 (59.9)	.315
Smoking status	. . .	. . .	. . .	.001	. . .	. . .	.022
Never smoked	455 (98.1)	204 (95.8)	251 (100.0)	. . .	286 (96.9)	169 (100.0)	. . .
Current/previous tobacco use	9 (1.9)	9 (4.2)	0 (0.0)	. . .	9 (3.1)	0 (0.0)	. . .
Smoking pack-y^a^	0.00 (0.00)	0.00 (0.00)	0.00 (0.00)	.001	0.00 (0.00)	0.00 (0.00)	.022
Duration of diabetes, y	10.00 ± 7.36	8.22 ± 7.38	11.58 ± 6.99	< .001	9.05 ± 7.25	11.68 ± 7.28	< .001
Insulin use	139 (30.0)	52 (24.4)	87 (34.7)	.016	76 (25.8)	63 (37.3)	.009
Statin use	192 (41.4)	62 (29.1)	130 (51.8)	< .001	102 (34.6)	90 (53.3)	< .001
Systolic BP, mm Hg	135.26 ± 15.67	131.90 ± 14.02	138.11 ± 16.44	< .001	133.22 ± 14.25	138.81 ± 17.36	< .001
Diastolic BP, mm Hg	79.14 ± 8.23	78.83 ± 7.46	79.41 ± 8.83	.444	79.08 ± 8.12	79.24 ± 8.44	.844
Pulse rate, beats/min	79.36 ± 10.72	78.00 ± 9.95	80.51 ± 11.24	.011	78.38 ± 10.44	81.05 ± 11.03	.010
Anthropometry							
BMI, kg/m^2^	29.13 ± 6.00	25.39 ± 3.63	32.30 ± 5.78	< .001	25.54 ± 2.97	35.39 ± 4.64	< .001
Waist circumference, cm	95.43 ± 11.88	86.92 ± 6.56	102.66 ± 10.52	< .001	89.70 ± 7.52	105.44 ± 11.47	< .001
Hip circumference, cm	106.84 ± 11.17	100.68 ± 6.98	112.07 ± 11.40	< .001	102.06 ± 7.42	115.20 ± 11.72	< .001
WHR	0.89 ± 0.07	0.87 ± 0.07	0.92 ± 0.07	< .001	0.88 ± 0.07	0.92 ± 0.07	< .001
Total body fat, %	38.29 ± 18.44	31.96 ± 26.40	42.66 ± 6.95	< .001	34.39 ± 21.60	45.15 ± 6.70	< .001
Visceral fat, %	11.12 ± 3.89	9.77 ± 3.82	12.05 ± 3.67	< .001	9.91 ± 3.52	13.24 ± 3.61	< .001
Muscle, %	27.69 ± 11.32	30.47 ± 5.38	25.77 ± 13.73	< .001	28.84 ± 5.17	25.66 ± 17.41	.010
Biochemical measurements							
HbA1c, %	7.98 ± 1.77	7.94 ± 1.91	8.00 ± 1.68	.735	7.99 ± 1.87	7.96 ± 1.62	.869
Total cholesterol, mmol/L	4.86 ± 1.27	5.02 ± 1.32	4.76 ± 1.24	.079	4.93 ± 1.30	4.75 ± 1.23	.227
Triglyceride, mmol/L	1.22 ± 0.50	1.24 ± 0.57	1.22 ± 0.46	.731	1.25 ± 0.57	1.20 ± 0.40	.348
HDL- cholesterol, mmol/L	1.34 ± 0.35	1.35 ± 0.35	1.33 ± 0.35	.618	1.35 ± 0.34	1.33 ± 0.35	.679
LDL-cholesterol, mmol/L	2.95 ± 1.15	3.11 ± 1.23	2.85 ± 1.10	.055	3.01 ± 1.21	2.86 ± 1.06	.249

Values are mean ± SD, No. (%), or as otherwise indicated. Obesity is defined as BMI ≥ 30 kg/m^2^; central obesity defined as WHR ≥ 0.90 for male participants and ≥ 0.85 for female participants. HDL = high-density lipoprotein; HBA1c = glycated hemoglobin; LDL = low-density lipoprotein; WC = waist circumference; WHR = waist-to-hip ratio.

a Expressed as median (interquartile range).

### Pulmonary Functional Impairments

The spirometric indices and pulmonary function measures in the two study groups are compared in Table 2. The mean FEV_1_ percent predicted and FVC percent predicted were, respectively, 10.6% lower (72.4% vs 81.0%, *P* < .001) and 11.1% lower (73.8% vs 83.0%, *P* < .001) in individuals with central obesity than in those without central obesity; the percent predicted FEV_1_/FVC ratio did not differ between the two groups. Similar results were obtained when *z* score FEV_1_, FVC, and FEV_1_/FVC ratios were compared between the two groups. When obesity was defined based on BMI ≥ 30 kg/m^2^, similar findings were observed; however, the difference in FEV_1_ percent predicted and FVC percent predicted between the two groups was less marked.

**Table 2:** Pulmonary Functional Measures in the Study Population

Variable	All Participants (N = 464)	Central Obesity (Based on WC)			Obesity (Based on BMI)		
		No (n = 213)	Yes (n = 251)	*P* Value	No (n = 295)	Yes (n = 169)	*P* Value
Percent predicted							
FEV_1_	76.31 ± 17.22	80.95 ± 18.38	72.38 ± 15.13	< .001	78.01 ± 17.64	73.35 ± 16.08	.005
FVC	78.03 ± 16.52	83.01 ± 16.50	73.80 ± 15.35	< .001	80.00 ± 16.42	74.58 ± 16.17	.001
FEV_1_/FVC ratio	98.04 ± 9.70	97.66 ± 10.23	98.35 ± 9.24	.445	97.75 ± 10.19	98.55 ± 8.79	.393
*z* score							
FEV_1_	−1.43 ± 1.02	−1.18 ± 1.12	−1.63 ± 0.87	< .001	−1.34 ± 1.06	−1.58 ± 0.92	.014
FVC	−1.39 ± 1.03	−1.11 ± 1.04	−1.63 ± 0.96	< .001	−1.28 ± 1.02	−1.59 ± 1.02	.002
FEV_1_/FVC	−0.16 ± 1.21	−0.21 ± 1.30	−0.12 ± 1.13	.437	−0.19 ± 1.28	−0.11 ± 1.07	.485
Pulmonary function pattern							
Normal spirometry	263 (56.7)	142 (66.7)	121 (48.2)	< .001	177 (60.0)	86 (50.9)	.057
Impaired spirometry^a^	201 (43.3)	71 (33.3)	130 (51.8)	< .001	118 (40.0)	83 (49.1)	.057
Restrictive spirometry	180 (38.8)	59 (27.7)	121 (48.2)	< .001	103 (34.9)	77 (45.6)	.024
Airway obstruction	35 (7.5)	19 (8.9)	16 (6.4)	.301	24 (8.1)	11 (6.5)	.523

Values are mean ± SD, No. (%), or as otherwise indicated. Obesity is defined as BMI ≥ 30 kg/m^2^; central obesity is defined as a waist-to-hip ratio ≥ 0.90 for male participants and ≥ 0.85 for female participants. Computation of percent predicted and *z* score spirometric indices were all based on the Global Lung Function Initiative equations using race-neutral average reference equations. WC = waist circumference.

a Impaired spirometry is defined as the presence of airway obstruction and/or lung restriction.

The proportion of individuals with spirometric impairments (restrictive spirometry and/or airway obstruction) was 56% higher in individuals with central obesity than in those without central obesity (51.8% vs 33.3%, respectively; *P* < .001). Although the proportion of individuals with restrictive spirometry was nearly 75% higher in individuals with central obesity compared with those without central obesity (48.2% vs 27.7%, respectively; *P* < .001), there was no significant difference between the two groups in terms of the proportion of individuals with airway obstruction. Similar but weaker associations were observed when obesity was defined based on BMI ≥ 30 kg/m^2^. For example, the proportion of individuals with restrictive spirometry was about 30% higher in individuals with BMI ≥ 30 kg/m^2^ than in those with BMI < 30 kg/m^2^ (45.6% vs 34.9%, respectively; *P* = .024).

### Associations Between Measures of Obesity and Pulmonary Dysfunction

**Figure 1:**
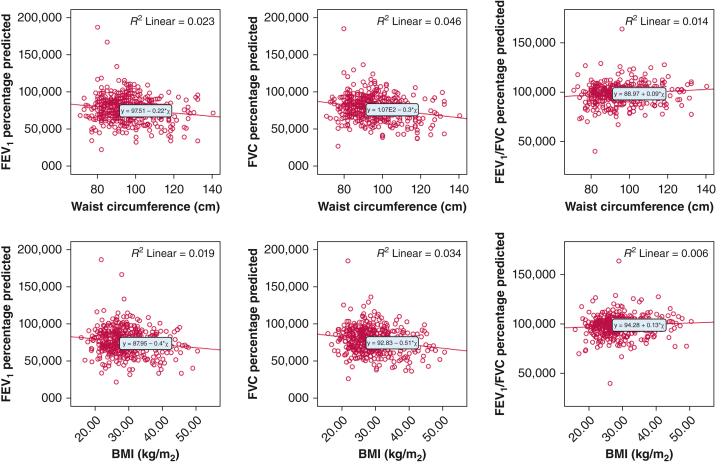
Associations of spirometric measures with BMI and waist circumference.

Figure 1 shows the linear associations of WC and BMI with FEV_1_, FVC, and FEV_1_/FVC ratio. There were significant and inverse correlations between WC and FEV_1_ percent predicted (*r* = −0.147, *P* = .002) and FVC percent predicted (*r* = −0.205, *P* < .001). There was a significant and positive correlation between WC and FEV_1_/FVC ratio (*r* = 0.171, *P* = .004). Similar observations were made for the associations of BMI with FEV_1_ percent predicted (*r* = −0.147, *P* = .002), FVC percent predicted (*r* = −0.196, *P* < .001), and FEV_1_/FVC ratio (*r* = 0.152, *P* = .010).

e-Table 1 summarizes the output of multiple linear regression models that sought to determine if there were linear relationships between spirometric measures and potential factors. WC was a significant linear predictor of FVC (*P* = .018) and FEV_1_/FVC ratio (*P* = .005), but not FEV_1_ (*P* = .472). Sex and diabetes duration were significant linear predictors of FEV_1_ and FVC, but not FEV_1_/FVC ratio. HbA1c was a significant predictor of FEV_1_, but not FVC or FEV_1_/FVC ratio. In a separate model where BMI was included (in place of WC), BMI was a significant predictor of FEV_1_/FVC ratio (*P* = .031), but not FEV_1_ (*P* = .802) or FVC (*P* = .129).

Table 3 shows the associations of *z* score WC and BMI with impaired spirometry. In the unadjusted model, increasing *z* score WC was associated with higher odds of restrictive spirometry (OR, 1.46; 95% CI, 1.20-1.77; *P* < .001). The positive association between *z* score WC and restrictive spirometry remained statistically significant in the age-adjusted and sex-adjusted model (OR, 1.32; 95% CI, 1.08-1.61; *P* = .006) and in the fully adjusted model (OR, 1.32; 95% CI, 1.05-1.66; *P* = .019). Increasing *z* score WC was inversely associated with the odds of airway obstruction in the unadjusted model (OR, 0.66; 95% CI, 0.45-0.99; *P* = .045) and the age-adjusted and sex-adjusted model (OR, 0.64; 95% CI, 0.43-0.95; *P* = .028), but not in the fully adjusted model (OR, 0.65; 95% CI, 0.42-1.03; *P* = .067). Increasing *z* score BMI was associated with higher odds of restrictive spirometry in the unadjusted model (OR, 1.44; 95% CI, 1.19-1.74; *P* < .001); the association was no longer statistically significant in the age-adjusted and sex-adjusted model (OR, 1.25; 95% CI, 1.02-1.53; *P* = .029) and the fully adjusted model (OR, 1.24; 95% CI, 0.98-1.58; *P* = .075). There were no significant associations between increasing *z* score BMI and airway obstruction in all three models.

**Table 3:** Logistic Regression Models for the Between Measures of Obesity (Independent Variable) and Pulmonary Dysfunction (Dependent Variable)

	Restrictive Spirometry			Airway Obstruction		
	Model 1	Model 2	Model 3	Model 1	Model 2	Model 3
*z* score WC	1.46 (1.20-1.77), < .001	1.32 (1.08-1.61), .006	1.32 (1.05-1.66), .019	0.66 (0.45-0.99), .045	0.64 (0.43-0.95), .028	0.65 (0.42-1.03), .067
*z* score BMI	1.44 (1.19-1.74), < .001	1.25 (1.02-1.53), .029	1.24 (0.98-1.58), .075	0.86 (0.60-1.24), .428	0.80 (0.55-1.18), .260	0.79 (0.51-1.24), .305

Values are OR (95% CI), *P* value. Model 1 is unadjusted. Model 2 is adjusted for age and sex. Model 3 is adjusted for age, sex, socioeconomic status, diabetes duration, glycated hemoglobin concentration, statin use, and smoking pack-years. Restrictive spirometry is based on measured FVC < lower limit of normal. Airway obstruction is based on FEV1/FVC < lower limit of normal. In computing the lower limit of normal for the spirometric indices, the Global Lung Function Initiative equations with race-neutral average reference equations were used. WC = waist circumference.

## Discussion

### Summary of Key Findings

This study that reports on the contribution of obesity to pulmonary dysfunction in T2D has four key findings. First, pulmonary functional impairment is prevalent in individuals with T2D without obesity. Obesity increases the odds of pulmonary dysfunction in T2D. Second, compared with BMI, WC is more strongly correlated with pulmonary dysfunction in T2D. Third, increasing WC is independently associated with restrictive spirometry. Finally, increasing WC is not independently associated with airway obstruction.

### Discussion of Key Findings

To our knowledge, this study is the first to compare spirometric impairments in individuals with T2D with and without obesity. Our study shows that in individuals with T2D without obesity, pulmonary dysfunction is prevalent. Respectively, 40.0% and 33.3% of individuals with T2D without obesity based on WC or BMI had pulmonary dysfunction, predominantly restrictive spirometry. These findings give credence to the view that pulmonary dysfunction in T2D occurs via pathways independent of obesity. Mechanistically, persistent hyperglycemia may destroy the alveolar epithelial cells or increase the production of proinflammatory and profibrotic factors, leading to pulmonary restriction.^28^ Alternatively, chronic hyperglycemia may result in nonenzymatic glycosylation of proteins in the lungs and thoracic cage, resulting in stiffening and reduction in lung and chest wall compliance.^9^

Although pulmonary dysfunction occurred in the absence of obesity, obesity significantly increased the odds of pulmonary dysfunction. For example, in a fully adjusted model, a unit SD increase in WC was associated with > 30% higher odds of restrictive spirometry. Our findings of a positive association between measures of obesity and pulmonary impairment agree with prior studies. For example, in individuals with metabolic disorders, WC is known to be an independent predictor of both FEV_1_ and FVC changes.^29^ Pulmonary function is also reported to be lower in individuals with metabolic syndrome (which has abdominal obesity as a component) and T2D.^30^ The relationship between obesity and impaired spirometry has also been previously reported in the general population, with a significant reduction in FVC occurring in individuals with obesity class 3, or when obesity coexists with another primary lung disease.^31^^,^^32^ In our study population, the proportion of individuals with obesity class 3 was remarkably low (5.6%). However, one in three individuals with obesity had a reduction in FVC below the lower limit of normal. This suggests that in T2D, a significant reduction in FVC occurs, without the need for obesity class 3 or the coexistence of another primary lung disease. Beyond the mechanical reduction in the chest wall and lung compliance in the setting of obesity, nonmechanical factors could contribute to the increased likelihood of pulmonary restriction in patients with T2D and obesity. A previous study that characterized respiratory function in mechanically ventilated patients with diabetes with and without associated obesity showed that diabetes alone and obesity alone resulted in similar alterations in pulmonary mechanical function, including an increase in airway resistance.^8^

In this study, we observed that compared with BMI, WC better correlated with pulmonary dysfunction in T2D. Although no prior study has compared the impact of central and general/peripheral obesity on pulmonary function in T2D, our findings agree with previous studies in the general population.^33^ Chen et al^33^ had previously reported that in the general population, WC, but not BMI, is negatively and consistently associated with pulmonary function in individuals with normal weight, who are overweight, and who are obese. Our observation of a stronger correlation between WC and pulmonary function compared with BMI and pulmonary function in T2D has a biological basis. Compared with BMI, WC is a better marker of central obesity. Central obesity, characterized by increased deposition of fat in the thorax, abdomen, and visceral organs, is more likely to have a direct adverse impact on pulmonary mechanics than peripheral obesity, which is characterized by the deposition of fat in the hips, thighs, and limbs. Existing reports also show that compared with peripheral obesity, central obesity has a greater impact on metabolic inflammation.^31^ Peripheral obesity is characterized by increased subcutaneous fat, whereas central obesity is characterized by increased visceral fat; visceral fat is more metabolically active than subcutaneous fat.^31^ For this reason, WC is also known to correlate more strongly to cardiovascular disease and mortality, compared with BMI.^34^ Our study suggests that WC instead of BMI should be considered when assessing the predictive role of obesity on pulmonary dysfunction in T2D.

Our findings of an independent association between increasing *z* score WC and higher odds of restrictive spirometry in T2D agree with previous reports in the general population. Wehrmeister et al^13^ reported inverse associations of WC with FEV_1_ and FVC. Our study that focuses on individuals with T2D adds to the existing literature in that we adjusted for a wide range of conventional risk factors. Our findings show that independent of conventional diabetes/pulmonary risk factors, abdominal obesity confers an additional risk of restrictive spirometry. Likely, decreased pulmonary compliance from abdominal obesity and/or diminished radial traction could be important mechanisms linking obesity to restrictive pulmonary defects in T2D.^35^ Compared with individuals without obesity, individuals with obesity tend to breathe at low lung volumes. At lower lung volumes, radial traction supporting the bronchi is lost, resulting in a smaller airway diameter.^35^ Breathing at smaller lung volume results in a narrow airway diameter, with an associated decrease in dynamic lung volumes including FEV_1_ and FVC; the FEV_1_/FVC ratio is however preserved.^36^

In this study, measures of obesity were not independently associated with airway obstruction in T2D. The relationship between measures of obesity and spirometric evidence of airway obstruction is more predictable in children. In children with asthma, both cross-sectional and longitudinal data show dysanapsis, in which FEV_1_ is reduced while FVC is increased, resulting in a lower FEV_1_/FVC ratio.^37^ In adults, the relationship is not that simple. Some studies have demonstrated an association between obesity with airway narrowing, and indicators of airway obstruction including incident asthma, asthma exacerbation, asthma severity, and poor asthma control.^38^ However, other studies show that individuals with obesity with or without coexisting obstructive airway diseases (eg, asthma) have normal spirometry or may have a marginal decline in FEV_1_ and FVC without a decline in the FEV_1_/FVC ratio.^35^^,^^39^ Obesity does not appear to confer an additional reduction in the FEV_1_/FVC ratio in individuals with asthma; however, it does affect asthma control.^39^ It is our considered view that the dual impact of obesity on restrictive spirometry and airway obstruction masks the spirometric evidence of airway obstruction (FEV_1_/FVC ratio < lower limits of normal) in individuals with obesity. This is especially true in T2D where the disease results in significant pulmonary restriction. Predominant restrictive spirometry (manifesting as a normal or elevated FEV_1_/FVC ratio) coexisting with less predominant airway obstruction (a reduction in the FEV_1_/FVC ratio) may tip the resultant FEV_1_/FVC ratio to values above the lower limit of normal. In conditions where early closure of the small airways occurs at low lung volumes, FVC may underestimate the slow vital capacity, resulting in preserved FEV_1_/FVC but a low FEV_1_/slow vital capacity ratio.^40^ Therefore, spirometric evidence (based on a reduced FEV_1_/FVC ratio) may not be the best option in assessing airway obstruction in individuals with obesity and T2D.

A surprising finding in this study was that both WC and visceral fat percentages were higher in the high BMI group than in the high WC group. Although the biological basis remains uncertain, it could reflect ethnic differences in the correlations between BMI, WC, and visceral fat.^41^ Data on associations between measures of adiposity in many ethnic groups including West Africans are limited.

### Limitations

Our study has limitations. First, the cross-sectional design limits the assessment of causation. Second, islet autoantibodies (eg, glutamic acid decarboxylase-65, insulin, tyrosine phosphatase 2, zinc transporter) were not measured in diagnosing T2D. Third, total lung capacity was not measured in assessing pulmonary restriction. However, when FEV_1_ and FVC are normal, omitting total lung capacity measurements overlooks only 4% of restrictive defects; this is usually in younger patients with sarcoidosis/nonspecific interstitial pneumonia.^42^ It is worth noting that although spirometry is useful for excluding pulmonary restriction, it is not able to accurately predict lung restriction. For example, one study showed that < 60% of patients with a classical spirometric restrictive pattern had pulmonary restriction confirmed on lung volume measurements.^43^ It is also possible that some of the study participants with restrictive spirometry have normal total lung capacity, in which case they would be considered to have an abnormal nonspecific pattern of pulmonary function tests.^44^ The nonspecific pattern has implications as to underlying physiology and etiology.^44^ Finally, the study did not assess potential confounders including insulin resistance, low-grade inflammation, and obesity hypoventilation syndrome because data on these were unavailable.

## Interpretation

Our study reports a relatively high prevalence of pulmonary functional impairment in individuals with T2D without obesity. However, obesity conferred a substantial additional risk for pulmonary functional impairment in T2D. Compared with BMI, WC was more strongly associated with pulmonary dysfunction in T2D. WC instead of BMI should be considered when evaluating the potential role of obesity on pulmonary dysfunction in T2D. Increasing WC was associated with restrictive spirometry, independent of conventional diabetes and pulmonary risk factors. Weight reduction could therefore improve pulmonary function in T2D. Health care providers need to encourage patients with known T2D with obesity and pulmonary dysfunction to prioritize weight loss as the initial approach to improving pulmonary function. Future research could explore the role of the reversal of central obesity on the reversal of pulmonary dysfunction in T2D.

## Funding/Support

This study was funded by a University of Ghana faculty development grant.

## Financial/Nonfinancial disclosures

None declared.

## Supplementary Data

e-Online Data
