# Supplementary material for: Pulmonary Function in Adults With Type 2 Diabetes With and Without Obesity
Source: CHEST Pulm. 2023 Sep 3;1(3):100014. doi: 10.1016/j.chpulm.2023.100014 (PMC13420462; doi:10.1016/j.chpulm.2023.100014)
Supplement: e-Online Data [file mmc1.docx]

**Supplementary Table 1: Multiple linear regression analyses for spirometric measures(outcome variable) and potential determinants.**

|  | **Beta (95% CI), p-value** | | |
| --- | --- | --- | --- |
|  | **FEV_1_ pp** | **FVC pp** | **FEV1/FVC pp** |
| ***WC Included in Model*** |  |  |  |
| Age | 0.05 (-0.08 to 0.25), 0.330 | 0.01 (-0.14 to 0.17), 0.831 | 0.06 (-0.06 to 0.17), 0.337 |
| Sex | 0.26 (5.51 to 12.79), <0.001 | 0.25 (4.92 to 11.79), <0.001 | 0.09 (-0.36 to 4.58), 0.094 |
| HbA1c | -0.10 (-1.74 to -0.04), 0.040 | -0.08 (-1.42 to 0.18), 0.127 | -0.05 (-0.87 to 0.28), 0.315 |
| Diabetes duration | -0.21 (-0.68 to -0.21), <0.001 | -0.22 (-0.66 to -0.22), <0.001 | -0.03 (-0.20 to 0.11), 0.590 |
| Smoking (current or previous) | -0.08 (-18.17 to 2.71), 0.146 | -0.07 (-16.86 to 2.86), 0.163 | -0.04 (-9.78 to 4.42), 0.459 |
| WC | -0.04 (-0.17 to 0.08), 0.472 | -0.12 (-0.26 to -0.02), 0.018 | 0.15 (0.04 to 0.21), 0.005 |
|  |  |  |  |
| ***BMI Included in Model*** |  |  |  |
| Age | 0.05 (-0.09 to 0.25), 0.344 | 0.01 (-0.15 to 0.17), 0.911 | 0.06 (-0.05 to 0.18), 0.280 |
| Sex | 0.26 (5.43 to 12.97), <0.001 | 0.24 (4.56 to 11.71), <0.001 | 0.11 (-0.13 to 5.02), 0.063 |
| HbA1c | -0.10 (-1.73 to -0.03), 0.041 | -0.08 (-1.42 to 0.19), 0.134 | -0.05 (-0.88 to 0.28), 0.312 |
| Diabetes duration | -0.21 (-0.68 to -0.22), <0.001 | -0.23 (-0.68 to -0.24), <0.001 | -0.02 (-0.19 to 0.13), 0.714 |
| Smoking (current or previous) | -0.07 (-17.99 to 2.88), 0.155 | -0.07 (-16.50 to 3.29), 0.190 | -0.04 (-10.07 to 4.18), 0.417 |
| BMI | -0.01 (-0.29 to 0.23), 0.802 | -0.08 (-0.44 to 0.06), 0.129 | 0.12 (0.02 to 0.37), 0.031 |

Abbreviations: BMI = body mass index, FEV_1_=forced expiratory volume in one second; FEV_1_/FVC = ratio of FEV_1_ to FVC, FVC = forced vital capacity; pp=percentage predicted; WC = waist circumference
